# Extending the Applicability of *In Ovo* and *Ex Ovo* Chicken Chorioallantoic Membrane Assays to Study Cytostatic Activity in Neuroblastoma Cells

**DOI:** 10.3389/fonc.2021.707366

**Published:** 2021-09-01

**Authors:** Miguel Angel Merlos Rodrigo, Berta Casar, Hana Michalkova, Ana Maria Jimenez Jimenez, Zbynek Heger, Vojtech Adam

**Affiliations:** ^1^Research Group for Molecular Biology and Nanomedicine, Department of Chemistry and Biochemistry, Mendel University in Brno, Brno, Czechia; ^2^Central European Institute of Technology, Brno University of Technology, Brno, Czechia; ^3^Instituto de Biomedicina y Biotecnología de Cantabria (IBBTEC), Consejo Superior de Investigaciones Científicas (CSIC) - Universidad de Cantabria, Santander, Spain; ^4^Centro de Investigación Biomédica en Red de Cáncer (CIBERONC), Instituto de Salud Carlos III, Madrid, Spain

**Keywords:** extracranial solid tumor, neuroblastoma, CAM assay, metastasis, preclinical trials, drug testing

## Abstract

**Purpose:**

The chick chorioallantoic membrane (CAM) assay can provide an alternative versatile, cost-effective, and ethically less controversial *in vivo* model for reliable screening of drugs. In the presented work, we demonstrate that CAM assay (*in ovo* and *ex ovo*) can be simply employed to delineate the effects of cisplatin (CDDP) and ellipticine (Elli) on neuroblastoma (Nbl) cells in terms of their growth and metastatic potential.

**Methods:**

The Nbl UKF-NB-4 cell line was established from recurrent bone marrow metastases of high-risk Nbl (stage IV, *MYCN* amplification, 7q21 gain). *Ex ovo* and *in ovo* CAM assays were optimized to evaluate the antimetastatic activity of CDDP and Elli. Immunohistochemistry, qRT-PCR, and DNA isolation were performed.

**Results:**

*Ex ovo* CAM assay was employed to study whether CDDP and Elli exhibit any inhibitory effects on growth of Nbl xenograft in *ex ovo* CAM assay. Under the optimal conditions, Elli and CDDP exhibited significant inhibition of the size of the primary tumor. To study the efficiency of CDDP and Elli to inhibit primary Nbl tumor growth, intravasation, and extravasation in the organs, we adapted the *in ovo* CAM assay protocol. In *in ovo* CAM assay, both studied compounds (CDDP and Elli) exhibited significant (*p* < 0.001) inhibitory activity against extravasation to all investigated organs including distal CAM.

**Conclusions:**

Taken together, CAM assay could be a helpful and highly efficient *in vivo* approach for high-throughput screening of libraries of compounds with expected anticancer activities.

## Introduction

Neuroblastoma (Nbl) is a heterogeneous pediatric cancer derived from the sympathetic nervous system arising from the neural crest (1). It is manifested as a solid malignant tumor residing around the spinal cord in the pelvis, chest, or neck and in the abdomen. Based on the statistics, Nbl accounts for ~15% of pediatric cancer deaths (2). While low- and intermediate-risk forms of Nbl are curable, patients with high-risk diseases have a 3-year event-free survival rate of only ~20% (3, 4), highlighting the utmost need for novel more efficient therapeutic agents.

Preclinical trials of novel anticancer therapeutic modalities require comprehensive *in vitro* and *in vivo* investigation. At the *in vivo* level, mouse xenograft models are the most common models employed to assess the drugs’ behavior (5). Noteworthy, rodent preclinical models are expensive and time-consuming and provoke ethical concerns. In contrast, the chick chorioallantoic membrane (CAM) assay is an efficient alternative to commonly used animal models (6, 7). Chick embryo is characterized by its essential immunodeficiency during its embryonic life, which is not typical for mice that frequently do not allow induction and growth of all types of human cancers (8). The CAM assay is a well-established *in vivo* system to study the angiogenesis and the carcinogenesis of various tumors, including prostate cancer (9), glioblastoma (10), osteosarcoma (11), colon cancer (12), non-small cell lung cancer (13), thyroid cancer (14), or Nbl (15). For these purposes, both *in ovo* (16) and *ex ovo* (17) CAM assays have been employed for various purposes varying in experiments from protocol to protocol. The benefit of the *ex ovo* CAM assay is that it enables a straightforward observation of angiogenesis, which is of utmost interest not only for microvascular research but also for screening of antiangiogenic drugs. On the other hand, *in ovo* CAM assay allows us easy monitoring of tumor extravasation and intravasation into the microvasculature and consequent formation of metastases in organs. Nevertheless, standardization of such protocols shows the main challenges and potential bottlenecks could be helpful to stimulate wider usage of CAM.

**Figure 1:**
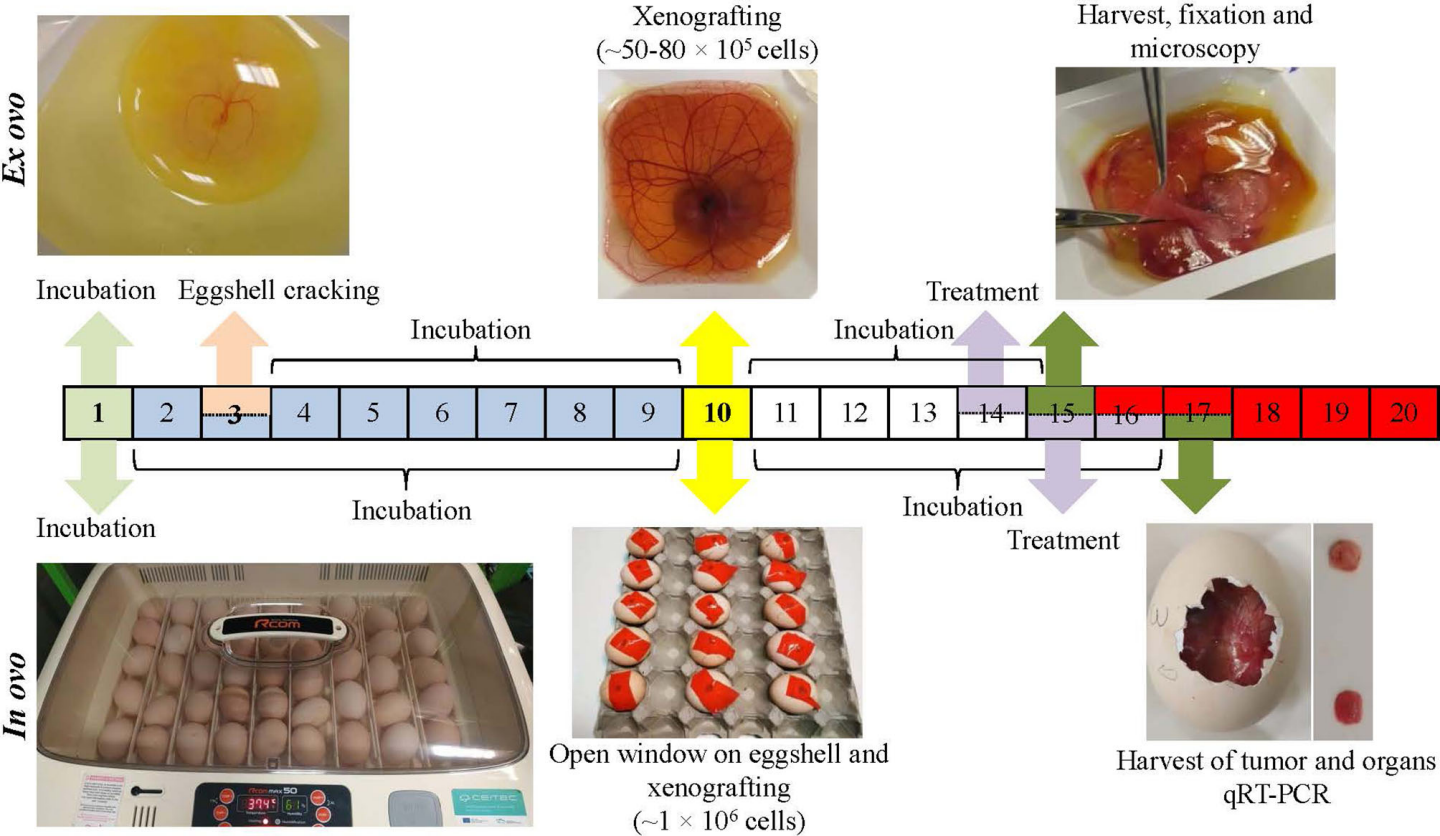
Workflow of *ex ovo* and *in ovo* chick chorioallantoic membrane (CAM) assays for development of neuroblastoma (Nbl) xenograft using UKF-NB-4 cells. For *ex ovo* CAM assay, the fertilized eggs were incubated for 3 days. Then, the eggshell was cracked, and the content was transferred into sterile dishes followed by incubation (additional 6 days). Nbl cells were then grafted on the CAM (specifically on vascular branches), and the embryos were incubated (additional 4 days). CAM areas with microtumors were extracted, keeping a 1-cm margin of healthy tissue, and were fixed. For *in ovo* CAM assay, the fertilized eggs were incubated for 10 days. In the eggshell, a window was opened, and Nbl cells were xenografted. The eggs were incubated for additional 6 days.

Herein, we provide a description and a detailed pipeline of development of both *in ovo* and *ex ovo* CAM models xenografted with human Nbl cells (**Figure 1**). The reported approach allows for a reliable production of 3-D, vascularized tumors histologically resembling undifferentiated Nbl. Importantly, we demonstrate that CAM is not only an *in vivo* model for investigation of angiogenesis and carcinogenesis, but also, as evidenced on the case study of conventional [cisplatin (CDDP)] and experimental [ellipticine (Elli)] cytostatics, it is an efficient model to study the activity of anticancer compounds. Without any doubt, the pipelined protocol can be thus utilized for high-throughput *in vivo* screening of candidate compounds to reveal the promising anticancer substances in a cost- and time-effective manner. Noteworthy, the use of CAM assay provides an efficient way to reduce and/or replace experiments on animals and could therefore accelerate development and preclinical testing of novel and more efficient therapeutic modalities, which could be interesting for various types of pathologies.

## Materials and Methods

### Chemicals

Chemicals were purchased from Sigma-Aldrich (St. Louis, MO, USA). The buffers and solutions were prepared with ACS purity chemicals unless noted otherwise.

### Cell Line and Culturing

The Nbl UKF-NB-4 cells were a generous gift from Prof. Tomas Eckschlager (Department of Pediatric Hematology and Oncology, 2nd Faculty of Medicine, Charles University in Prague and Motol University Hospital, Prague, Czechia). UKF-NB-4 cells were established from recurrent bone marrow metastases of high-risk Nbl (stage IV, *MYCN* amplification, 7q21 gain). The cells were cultured in Iscove’s modified Dulbecco’s medium (IMDM) with 10% fetal bovine serum and grown at 37°C and 5% CO_2_. The cells were passaged at regular intervals twice a week.

### *Ex Ovo* Chorioallantoic Membrane Assay

The fertilized chicken eggs purchased from a local provider (Vykrm Trebic, Trebic, Czechia) were incubated lying horizontally with intermittent rotation at 37.5°C and 65% humidity for 3 days. Then, the eggshells were cracked, and embryos were transferred into small, sterile plastic bowls (VWR, Radnor, PA, USA). During the transfer process, viability of the embryos was monitored, and the developing embryo must be always placed on the top of the yolk sac. Then, the bowls were covered using Petri dishes and incubated for 7 days more after which embryos were xenografted (four to six different sites that were always near to small vessels) with 5 × 10^4^ cells/25 µl of serum-free medium always close to small vessels, and the site of inoculation was labeled on a Petri dish. The embryos’ survival rates were checked on a daily basis. Before xenografting, UKF-NB-4 cells were pre-labeled with CellTracker Green (Invitrogen, Carlsbad, CA, USA), mixed with Geltrex LDEV-Free reduced growth factor basement membrane matrix (Invitrogen, Carlsbad, CA, USA), and implanted on the CAM at an initial seeding density of ~5 × 10^4^. After 3 days of incubation, 5 µl of 100 μM CDDP or 200 μM Elli in phosphate-buffered saline (PBS) was administered directly onto each microtumor, and the embryos were left without movement for 30 min to enable absorption of CDDP/Elli to the microtumor and to avoid possible runoff from the microtumor sites. Then, *ex ovo* cultures were further incubated at 37.5°C for 24 h. For the purpose of fluorescence angiography, vascular endothelium was labeled with rhodamine-labeled *Lens culinaris* agglutinin (LCA; 50 μl of 5 μg/ml) (Vector Laboratories, Burlingame, CA, USA) administered in peripheral vein with 30-gauge hypodermic needle. Upon this procedure, the embryos were further incubated (3 min) to enable LCA to circulate and stain vascular endothelium. Then, by cutting the vitelline arteries, the embryos were sacrificed. CAM sites that produced microtumors were extracted with a 1-cm margin around microtumors and fixed using 3.7% paraformaldehyde in PBS. For subsequent fluorescence angiography, the confocal laser scanning microscopy (CLSM) (LSM 880, Carl Zeiss, Jena, Germany) was used with the emitted light from rhodamine collected in a detection window 570–580 nm, and green light from UKF-NB-4 cells was labeled with CellTracker Green collected at 515–525 nm.

### *In Ovo* Chorioallantoic Membrane Assay

*In ovo* CAM assay to determine the antimetastatic activity of CDDP and Elli followed the protocol described by Crespo et al. (18). Briefly, fertilized chicken eggs were incubated with intermittent rotation (37.5°C, 65% humidity, 10 days). On day 10, eggs were put on a rack; and a hole was drilled in the air sac at the blunt end of the egg using a 30-gauge syringe. Further, a Dremel rotary tool kit (Dremel, Racine, WI, USA) was employed to drill another hole near the allantoic vein while using a light source to control the drilling and to avoid causing an injury to the CAM. After that, another hole was drilled with a 20-gauge syringe needle and a mild vacuum was applied to the air sac hole using an automated pipette with a Tygon tube to drop the CAM from the shell. Further, a square window (~1 cm^2^) was drilled with a cut-off wheel (Dremel) close to the bifurcation of the allantoid vein. Then, 25 µl of the suspension of ~1 × 10^6^ UKF-NB-4 cells in the serum-free medium was applied close to the allantoid vein bifurcation using a pipette while avoiding a direct contact with the CAM. After xenografting, the eggs were left standing upright for 5–10 min in order to allow the cells to settle, sealed with tape, and left to grow (6 days at 37.5°C) on a stationary incubator. Then, 100 µM of CDDP or 200 µM of Elli in PBS was added topically directly onto each microtumor on the upper CAM and the eggs were left without movement for 30 min to enable absorption of CDDP/Elli to the microtumor and to avoid possible runoff from the microtumor sites. After that, the eggs were incubated for additional 24 h according to our previous study (15). At defined time-points, parts of the CAM, liver, lung, and brain were extracted to carry out additional analyses and to quantify the amount of human tumor cells that accumulated in the tissues using qPCR. In the European Union countries, CAM assay is not considered an animal experiment and, therefore, does not require ethical approval.

### Isolation of DNA and qRT-PCR

Genomic DNA from tissues was extracted using DNeasy Blood & Tissue kit (Qiagen, Germantown, MD, USA). Before qPCR, the DNA was diluted 1:50 or 1:25 to get 30 ng/µl in nuclease-free water. To quantify human tumor cell DNA in the extracted tissues, we employed qPCR quantifying human *Alu* sequences by utilizing *Alu*-specific primers and SYBR green mix amplification kit. Reaction mix was prepared with 0.4 μM of each primer in a final volume of 10 μl. Chicken GAPDH primers were utilized as internal control. The qPCR conditions were as follows: 95°C for 2 min, 40 cycles at 95°C for 30 s, 63°C for 30 s, and 72°C for 30 s. From the UKF-NB-4 cell DNA, standard curve was generated using a dilution series (10^2^, 10^3^, and 10^4^). The standard curve was subsequently utilized to quantify human tumor cells in chick tissues through Ct values obtained in triplicates. To statistically evaluate results, Student’s *t*-test or ANOVA was carried out in GraphPad Prism (GraphPad, San Diego, CA, USA). The specificity of the qPCR reactions was verified by melting curve analysis. Primer design was done using the genes sequences available at the NCBI GenBank (www.ncbi.nih.gov). The following primers were custom designed and synthesized by Sigma-Aldrich: *Alu*(human)Fw: 5′-ACGCCTGTAATCCCAGGACTT-3′ *Alu*(human)Rv: 5′-TCGCCCAGGCTGGCTGGGTGCA-3′; GAPDH(*chicken*)Fw: 5′-GAGGAAAGGTCGCCTGGTGGATCG-3′ and GAPDH(*chicken*)Rv: 5′ GGTGAGGACAAGCAGTGAGGAACG 3′.

### Immunohistochemistry

After extraction, tissues were embedded in paraffin and cut using microtome; and 5-µm sections were placed on poly-l-lysine-treated slides. Before staining, slides were deparaffinized, and tissues were rehydrated. After that, slides were dried (1 h at 60°C or overnight at 37°C). Then, permeabilization of cells was carried out by incubation (10 min) of tissues with 0.1% IGEPAL in 1× Tris Buffered Saline (TBS). After that, the specimens were washed (2×, 5 min with 1× TBS), and non-specific bindings were blocked using a serum-free blocking agent, background punisher (BIOCARE Medical, Pacheco, CA, USA), for 8 min. Then, 1% bovine serum albumin (BSA) 0.05% IGEPAL in 1× TBS solution with a primary mouse anti-rat CD44 antibody (diluted 1:100) (Antibodies Online, Aachen, Germany) or without primary antibody (negative control) was incubated overnight (4°C). After incubation, specimens were washed in 1× TBS and incubated with 3× hydrogen peroxide in 1× TBS for 20 min to quench endogenous peroxidase. After that, the slides were washed again using 1× TBS and incubated (1 h) with a secondary anti-mouse biotinylated antibody (Vector Laboratories, Burlingame, CA, USA) (dilution 1:400 in 1% BSA 0.05% IGEPAL in 1× TBS). Then, slides were washed, as described above, and tissues were incubated (30 min) with horseradish peroxidase avidin D (dilution 1:500 in 1× TBS). Finally, slides were washed again and incubated (5 min) with diaminobenzidine (Gibco, Gaithersburg, MD, USA). In addition, tissues were stained with hematoxylin, dehydrated, cleared, and mounted with DPX mountant. Micrographs were captured by Zeiss Axio Scope A1 microscope (Carl Zeiss).

## Results

### *Ex Ovo* Chorioallantoic Membrane Assay Examining the Effect of Cisplatin and Ellipticine

**Figure 2:**
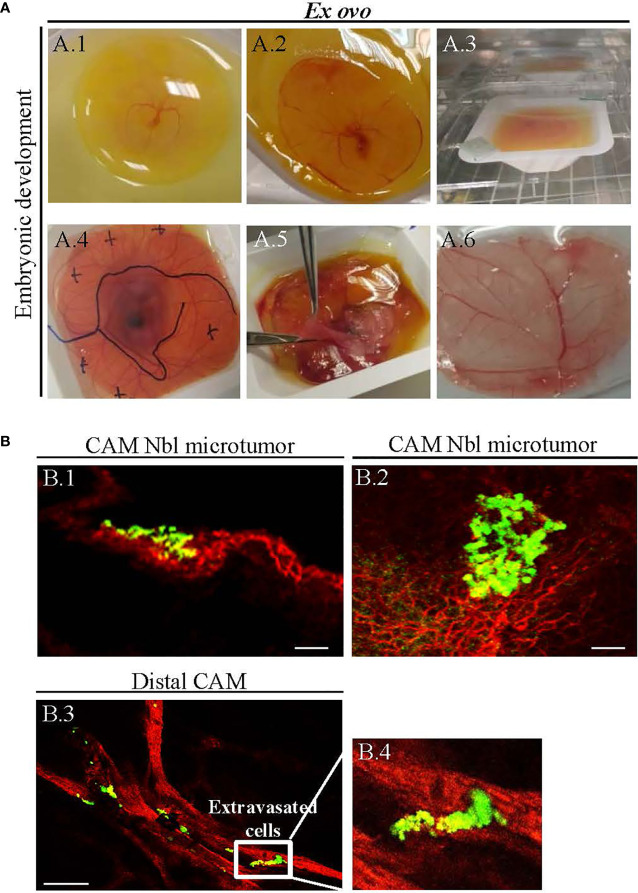
**(A)***Ex ovo*-cultivated chicken embryos exhibit physiological development over time. **(A.1)** Successful transfer of embryo having an intact egg yolk (on embryonic development day 3), and **(A.2–A.4)** live embryo on embryonic development from day 3 to 10. **(A.4)** *In vitro*-cultured neuroblastoma (Nbl) cells were transferred on the chick chorioallantoic membrane (CAM) at day 10, and the sites of xenografting were marked on the cover of the Petri dish. **(A.5)** Harvesting of Nbl tumors from CAM at day 14. **(A.6)** The CAM fixation for microscopy. **(B)** Confocal laser scanning microscopy (CLSM) validation of successful establishment of UKF-NB-4 xenografts on CAM. **(B.1, B.2)** Fluorescence angiography showing Nbl microtumors (green, labeled with CellTracker) surrounded by extensive amount of blood vessels [red, labeled with rhodamine *Lens culinaris* agglutinin (LCA)]. **(B.3, B.4)** Invasive vasculotropic Nbl cells that escaped from primary tumor site and extravasated at the distal CAM. Scale bars, 200 μm.

In this study, we adapted the conditions of *ex ovo* CAM previously reported by Herrero et al. (19) and Casar et al. (20). Use of this methodology allows the survival of embryos to be >50%. Established *ex ovo* CAM assay was further employed to study whether CDDP and Elli exhibit any inhibitory effects on growth of Nbl xenograft in *ex ovo* CAM assay. **Figure 2** shows that on embryonic development day 3, embryo was transferred successfully with an intact egg yolk (**Figure 2A1**), and live embryo was on embryonic development from day 3 to 10 (**Figures 2A2–A4**). At day 10 of embryonic development, Nbl cells were xenografted on the CAM and the sites of inoculation were labeled on Petri dish covering the bowl with *ex ovo*-incubated embryo (**Figure 2A4**). At day 14, the Nbl cells forming microtumors were harvested (**Figure 2A5**), and the whole CAM was processed for subsequent analyses (**Figure 2A6**).

**Figure 3:**
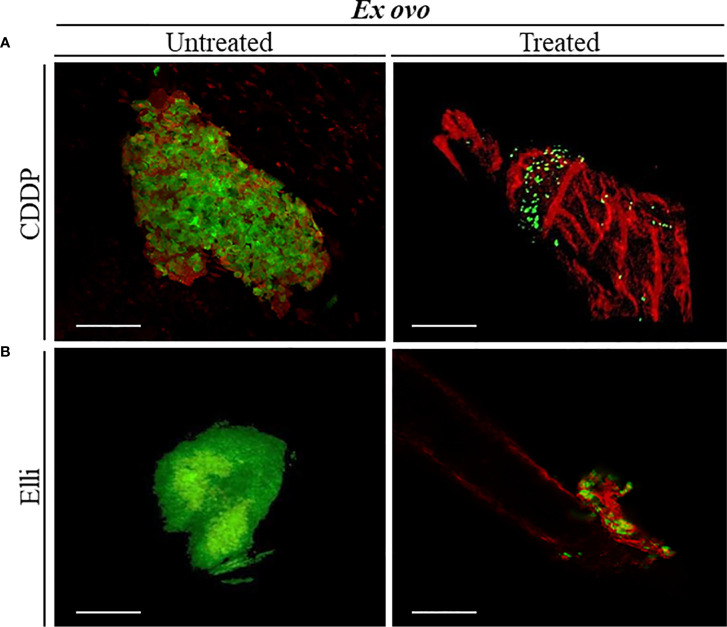
Examination the inhibitory effects of **(A)** cisplatin (CDDP) and **(B)** ellipticine (Elli) on neuroblastoma (Nbl) tumor development by *ex ovo* chick chorioallantoic membrane (CAM) assay. Confocal laser scanning microscopy (CLSM) micrographs of UKF-NB-4 tumor treated with CDDP and Elli for 24 h (each photo corresponds to an independent tumor). Viable Nbl cells are green (labeled with CellTracker), and angiogenic vessels are red [labeled with rhodamine *Lens culinaris* agglutinin (LCA)]. Scale bar, 200 μm.

**Figures 2B.1, B.2** demonstrate that UKF-NB-4 xenografts were established correctly on CAM as evidenced by extensive green signal of CellTracker-labeled Nbl cells. Importantly, the vasculature of embryo and Nbl cells can be observed concurrently to evaluate a process of the formation of metastasis including the cell extravasation towards the distal CAM (**Figures 2B.3, B.4**). Moreover, UKF-NB-4 tumors administered with Elli and CDDP displayed a marked reduction of the size of the primary tumor in the *ex ovo* CAM assay. As shown in **Figure 3**, after 24 h of exposure to CDDP or Elli, the UKF-NB-4 microtumors disappeared completely or mostly, and no evidence of Nbl cells intravasation and extravasation was found. This highlights the reliability of *ex ovo* CAM assay for *in vivo* screening of compounds with expected anticancer activity.

### *In Ovo* Chorioallantoic Membrane Assay Determining the Effect of Cisplatin and Ellipticine

**Figure 4:**
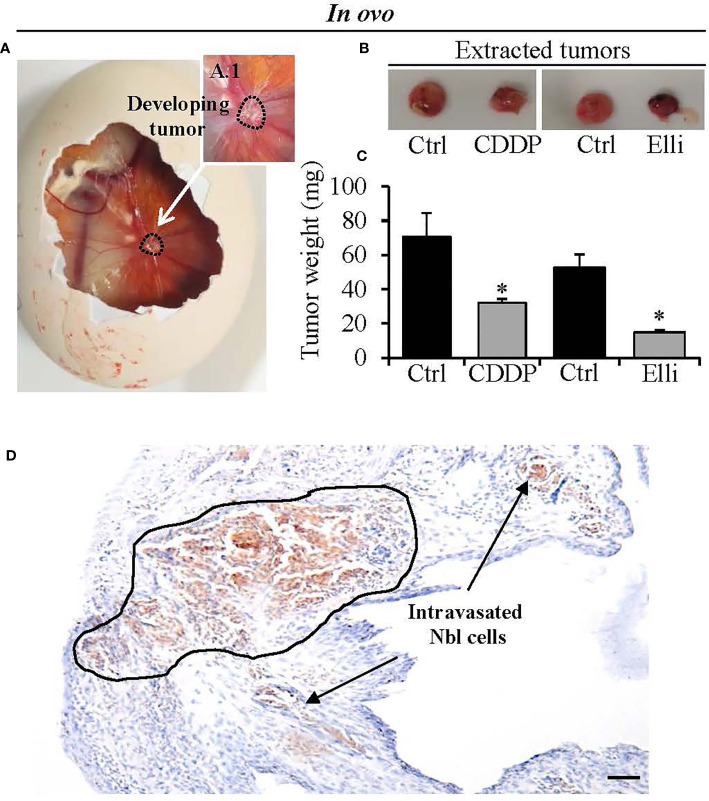
Determination of efficiency of treatment [cisplatin (CDDP) and ellipticine (Elli)] to inhibit growth of neuroblastoma (Nbl) xenograft. **(A)** *In ovo* chick chorioallantoic membrane (CAM) assay photograph showing representative CAM with the Nbl tumor formed at the seventh day after xenografting. **(B)** Photographs and **(C)** tumor weights after excision from the CAM (upon experiment termination on the 17th day). **(D)** Immunohistochemical visualization of CD44 expression in Nbl cells forming primary tumor and outgrowing from primary tumor by disrupting the CAM upper epithelium (black demarcation). Intravasated Nbl cells in the distal CAM are shown by arrows. Data show mean ± SEM from three (*n* = 3) independent experiments. *p < 0.001. Scale bar, 200 μm.

To study the efficiency of CDDP and Elli to inhibit primary Nbl tumor growth, intravasation, and extravasation in the organs, we adapted the *in ovo* CAM assay protocol previously published by Crespo et al. (18). Importantly, by using this approach, within 7 days from induction, we achieved 100% (20/20) embryo survival, and all of them had successfully developed Nbl tumors on CAM (**Figures 4A, A1**). Upon termination, tumor growth was assessed through weight measurements, confirming significant (*p* < 0.001) inhibitory cytostatic activity of CDDP (approx. two-fold decline of primary tumor weight) and Elli (approx. 3.5-fold decline of primary tumor weight) after 24 h (**Figures 4B, C**). By further immunolabeling of CD44, a formed primary tumor in CAM can be simply visualized, and its margin can be demarcated as shown in **Figure 4D**. It was found that the Nbl cells grow from the primary tumor site to the neighboring CAM, thus disrupting the upper epithelium of CAM. In addition, **Figure 4D** clearly shows intravasated cells in distal CAM.

**Figure 5:**
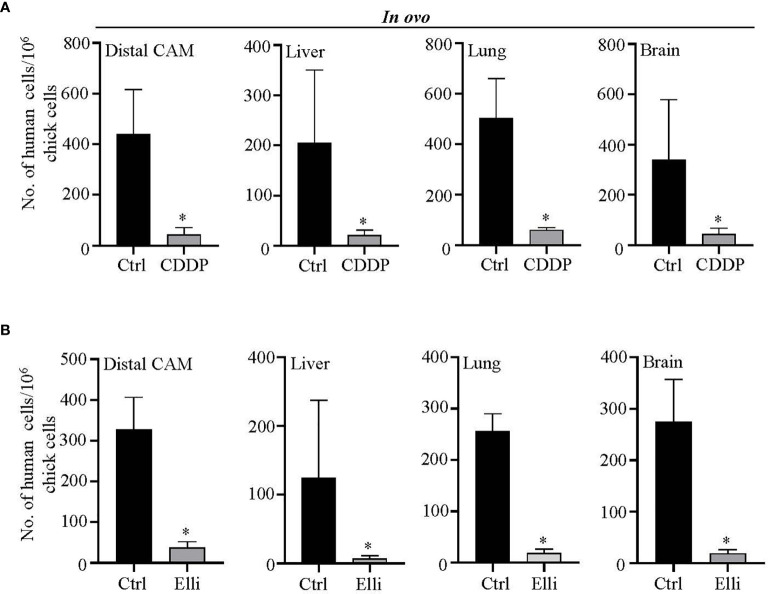
Evaluation of efficiency of **(A)** cisplatin (CDDP) and **(B)** ellipticine (Elli) to inhibit metastatic spread of UKF-NB-4 cells to distal chick chorioallantoic membrane (CAM) and selected organs. qPCR quantitation of human (neuroblastoma (Nbl)) cells using universal *Alu* primers. As internal control used to validate the presence of equivalent amount of host (chicken) genomic DNA, we employed chicken GAPDH primers. Data show mean ± SEM from three (*n* = 3) independent experiments. *p < 0.001.

Another advantageous feature of CAM is that it allows us simple quantitation of intravasated/extravasated cells through qPCR with universal human *Alu* primers that only amplify the human sequences, while avoiding the amplification of DNA of host organism. By this approach, we further investigated the amounts of Nbl cells forming a metastatic spread to different organs (the liver, lung, brain, and distal CAM). As shown in **Figures 5A, B**, in *in ovo* CAM assay, both studied compounds (CDDP and Elli) exhibited significant (*p* < 0.001) inhibitory activity against extravasation to all investigated organs including distal CAM. Noteworthy, such results are most likely caused by a high cytostatic activity of relatively high concentrations (but not high enough to kill the host organism) of both compounds, resulting in a rapid and efficient arrest of cell growth and induction of cell death rather than caused by their antimetastatic activity. Taken together, the obtained data clearly show that CAM assay could be a helpful and highly efficient *in vivo* approach for high-throughput screening of libraries of compounds with expected anticancer activities.

## Discussion

This study aimed to highlight the importance of CAM assay for evaluation of new treatment options relevant for high-risk Nbl. Nevertheless, it must be noted that the presented methodologies can be simply extended to any other solid cancer type with only one major adjustment: optimization of cancer cell amounts to develop CAM tumors. The advantages and limitations of the CAM assay as a versatile *in vivo* model have been well-described with a particular emphasis on research in the field of angiogenesis and carcinogenesis (21, 22). In terms of drug testing, the majority of available studies utilize CAM assay to study antiangiogenic or angiogenic potential of examined compounds/materials and their consequent effect on the development of embryo (23, 24). However, few available studies have focused on the use of CAM assay to assess invasiveness and metastatic potential of Nbl cells (25–27), and none of them employed CAM assay to investigate the anticancer compounds. The *ex ovo* variant of the classical *in ovo* CAM assay offers several unique advantages for drug testing. However, it has been reported by several authors that the *ex ovo* CAM assay is associated with worse survival rates (17, 28). This is in line with our previous experiments during which the survival of embryos in *ex ovo* CAM assay is between 50% and 70%. The main advantage of the *ex ovo* CAM procedure is facile visualization of the growing embryo and access to a larger area of the CAM to study size of tumors and migration of cells into the vessels (8). Besides, *in ovo* CAM assay allows easy monitoring of tumor extravasation and intravasation into the microvasculature and consequent formation of metastases in organs evaluated by qPCR. Therefore, we provide the first condensed pipeline demonstrating applicability of CAM assay (*in ovo* and *ex ovo*) as a facile and efficient tool to study the activity of candidate molecules for anticancer therapy *in vivo* without need of rodent facility and associated ethical approvals and concerns. Besides, the CAM is not innervated so that experiments are not associated with pain perception by the embryo, and there is no need for ethical approval for animal experimentation. Current laws regulating animal experimentation in the European Union and Switzerland allow experimentation with chick embryos without authorization from animal experimentation committees, on the grounds that experiments begin and end before hatching (until day 17) (29, 30). This could be of utmost interest for screening purposes as well as for those experiments not depending on animal facility.

Among the main causes of deaths of children suffering from Nbl are metastasis and tumor resistance to conventional treatment (31). A substantial progress of understanding of Nbl biology has been made in the past decades. This resulted in a number of clinical trials that have been undertaken to find new therapeutic options for high-risk Nbl management (32, 33). During the metastatic progression, Nbl cells must proceed through a complex process, which includes invasion, intravasation, extravasation, and colonization of the target organs (34). In the presented study, we have shown that by combining both *in ovo* and *ex ovo* approaches, CAM assay can be employed to comprehensively study these processes *in vivo* either when administered with cytostatic drug or non-treated. Indeed, our results point to the remarkable efficacy of CDDP, which is a backbone of a first-line therapy of a broad spectrum of malignant diseases including metastatic ones (35). We also confirmed the interesting anticancer activity of Elli, which is a frequently investigated candidate compound suffering from low bioavailability and a plethora of side effects (36). Importantly, Elli is a perfect example of an imperfect lead compound that could be employed for development of novel derivatives with improved pharmacokinetic and toxicological activities. These can be investigated in CAM assay in a high-throughput regime. We are fully aware that screening of drugs using CAM assay does not have the potential to fully avoid the use of rodent experimental models. However, we anticipate that inclusion of CAM assay to the experimental pipelines of large-scale *in vivo* testing could result in a faster and cheaper revelation of compounds with a real therapeutic potential.

It can be concluded that we show that the applicability of CAM assay can be simply extended from its primary purpose to study carcinogenesis and angiogenesis to the field of screening of anticancer activity of cytostatic substances. By combining *in ovo* and *ex ovo* approaches, an array of crucial data regarding the effect of tested compound on the growth of primary tumor, and subsequent intravasation and extravasation of cancer cells to the distal organs can be gathered in a simple and cheap way without any ethical concerns. It must be noted that the applicability of CAM extends the investigated phenomena and the *in vivo* nature of CAM assay also enables a detailed molecular or biochemical examination of extracted tissues to fully delineate the effect of drug administration on the exposed organism.

## Data Availability Statement

The original contributions presented in the study are included in the article/supplementary material. Further inquiries can be directed to the corresponding authors.

## Author Contributions

All authors contributed to the study conception and design, MM and BC carried out most of the experimental work and inspected and manually corrected raw data. BC and HM participated in microscopy and immunohistochemistry experiments. AM carried out qPCR analyses. ZH participated in the study design, coordinated the execution of measurements, and corrected the working version of the manuscript. MM and VA designed the study, coordinated the work, wrote the manuscript, and are the corresponding authors. All authors contributed to the article and approved the submitted version.

## Funding

The authors acknowledge funding from the European Research Council (ERC) under the European Union’s Horizon 2020 Research and Innovation Programme (grant agreement No. 759585), the Czech Science Foundation (project no. 19-13766J), FEBS - Federation of European Biochemical Societies, and under the CEITEC 2020 project (LQ1601) by the Ministry of Education, Youth and Sports of the Czech Republic. BC is funded by Ramón y Cajal Research Program Ministry of Science and Innovation of Spain (MICIN, RYC2018-024004-I).

## Conflict of Interest

The authors declare that the research was conducted in the absence of any commercial or financial relationships that could be construed as a potential conflict of interest.

## Publisher’s Note

All claims expressed in this article are solely those of the authors and do not necessarily represent those of their affiliated organizations, or those of the publisher, the editors and the reviewers. Any product that may be evaluated in this article, or claim that may be made by its manufacturer, is not guaranteed or endorsed by the publisher.
